# Protection from T cell-dependent colitis by the helminth-derived immunomodulatory mimic of transforming growth factor-β, *Hp*-TGM

**DOI:** 10.1093/discim/kyad001

**Published:** 2023-01-18

**Authors:** Danielle J Smyth, Madeleine P J White, Chris J C Johnston, Anne-Marie Donachie, Marta Campillo Poveda, Henry J McSorley, Rick M Maizels

**Affiliations:** Wellcome Centre for Integrative Parasitology, School of Infection and Immunity, University of Glasgow, Glasgow, UK; Division of Cell Signalling and Immunology, University of Dundee, Dundee, UK; Wellcome Centre for Integrative Parasitology, School of Infection and Immunity, University of Glasgow, Glasgow, UK; Department of Clinical Surgery, University of Edinburgh, Edinburgh, UK; Wellcome Centre for Integrative Parasitology, School of Infection and Immunity, University of Glasgow, Glasgow, UK; Wellcome Centre for Integrative Parasitology, School of Infection and Immunity, University of Glasgow, Glasgow, UK; Division of Cell Signalling and Immunology, University of Dundee, Dundee, UK; Wellcome Centre for Integrative Parasitology, School of Infection and Immunity, University of Glasgow, Glasgow, UK

**Keywords:** dextran sodium sulphate, *Heligmosomoides polygrus*, inflammatory bowel disease

## Abstract

In animal models of inflammatory colitis, pathology can be ameliorated by several intestinal helminth parasites, including the mouse nematode *Heligmosomoides polygyrus*. To identify parasite products that may exert anti-inflammatory effects *in vivo*, we tested *H. polygyrus* excretory–secretory (HES) products, as well as a recombinantly expressed parasite protein, transforming growth factor mimic (TGM), that functionally mimics the mammalian immunomodulatory cytokine TGF-β. HES and TGM showed a degree of protection in dextran sodium sulphate-induced colitis, with a reduction in inflammatory cytokines, but did not fully block the development of pathology. HES also showed little benefit in a similar acute trinitrobenzene sulphonic acid-induced model. However, in a T cell transfer-mediated model with recombination activation gene (RAG)-deficient mice, HES-reduced disease scores if administered throughout the first 2 or 4 weeks following transfer but was less effective if treatment was delayed until 14 days after T cell transfer. Recombinant TGM similarly dampened colitis in RAG-deficient recipients of effector T cells, and was effective even if introduced only once symptoms had begun to be manifest. These results are a promising indication that TGM may replicate, and even surpass, the modulatory properties of native parasite HES.

## Graphical Abstract

**Graphical Abstract:**
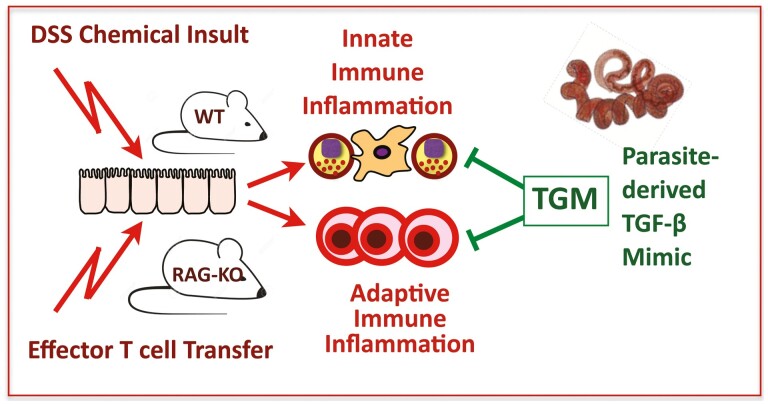

## Introduction

Inflammatory bowel diseases (IBDs) are immunological disorders with increasing prevalence in humans and limited therapeutic options [1–4]. Inflammation is largely mediated by T-cell subsets reacting to intestinal antigens such as those presented from commensal microorganisms and is attributed to dysregulation of the natural control mechanisms that prevent inappropriate immune responses to self- and environmental antigens. While the cause of such dysregulation has yet to be determined, treatment strategies that boost natural regulatory pathways may offer transformational new therapies for IBD [5, 6].

Among many drivers of immune regulation, helminth parasites have engendered much interest as they have adapted over long evolutionary time to target and dampen the immune system of their host [7, 8]. In the setting of colitis, a range of helminths—in particular those involving the gastrointestinal tract—have been found to alleviate inflammation in rodent models [9–12], and there is epidemiological evidence that helminth-infected children are less prone to develop IBD [13] In a placebo-controlled primate study, macaques treated with the pig whipworm *Trichuris suis* were protected from an idiopathic form of colitis [14]. However, similar trials in IBD patients infected with whipworms have fallen short of expectations [15, 16]. Although more promising results were found in Coeliac disease patients given the human hookworm *Necator americanus* [17], concerns of limited efficacy and practicality of live helminth infections have led to greater emphasis on molecular mediators derived from helminths that could be configured into pharmacological products for IBD therapy [18–20].

One route to discovering anti-inflammatory products from helminths is through mouse models in which therapeutic candidates can be readily identified and tested for efficacy in a homologous system. An ideal such model is *Heligmosomoides polygyrus*, a natural murine intestinal parasite related to the human hookworms, which is known to alleviate colitis in a range of experimental settings [21–24]. The immunomodulatory activity of parasite infection can be recapitulated in many respects by molecules released by adult worms *in vitro* (the ‘Excretory-Secretory’ or ES products), that have been well characterized in *H. polygyrus*. One objective of the current study was to ascertain if *H. polygyrus* ES (HES) products could protect mice from colitic inflammation.

Within the complex of proteins that are present in HES, we recently identified a novel protein secreted by *H. polygyrus* which acts as a functional mimic of TGF-β, which we have designated as transforming growth factor mimic (TGM). Despite no sequence similarity to the mammalian cytokine, TGM binds to host TGF-β receptors and like TGF-β itself, activates the pathway that induces regulatory T cells (Tregs) [25–28]. TGF-β signalling is known to play a critical role in controlling intestinal inflammation [29–31] and indeed recently proposed therapeutic for IBD sought to enhance TGF-β activation by inhibiting its negative regulator Smad7, an inhibitor of this pathway [32]. Moreover, activation or expansion of the Tregs compartment represents a potential therapeutic strategy for IBD [5].

In recent studies, we have shown that TGM can mediate suppression of immune inflammation, e.g. in models of airway allergy [33]. We also established the ability of TGM to abate the severity of dextran sodium sulphate (DSS) colitis in mice, using high doses delivered orally through admixture with drinking water [34]. Focusing on colitis, we now evaluate in more depth the relative potencies of HES and TGM, using several different murine models of colitis [35], including inflammation induced with trinitrobenzene sulphonic acid (TNBS), and a T cell transfer model in which adaptive immune responses to intestinal antigens more closely represents human disease.

## Materials and methods

### Animals

Transgenic mouse strains (all on C57BL/6 background) RAG1^−/−^ [36] and Foxp3-GFP [37] for *in vivo* experiments were aged 6–12 weeks old and bred in-house or purchased from Envigo (UK). BALB/c mice for TNBS colitis experiments were bred in-house. No randomization was used in setting up experiments. Animals were housed in individually ventilated cages. All transgenic animal breeding and procedures were approved by the University ethics committees and performed under UK Home Office licence, and all research adhered to the ARRIVE guidelines.

### Generation of immunomodulators

HES was prepared by culturing adult worms in protein-free media and diafiltration into phosphate-buffered saline (PBS) as described in detail elsewhere [38]. Only HES batches with bacterial endotoxin lipopolysaccharide (LPS) measuring below 1 U/µg of protein were used for experiments, measured using the endpoint chromogenic limulus amebocyte lysate assay (Lonza). Recombinant mammalian codon-optimized *H. polygyrus* TGM (corresponding to TGM-1 in the gene family described by Smyth *et al.* [39] was expressed using mammalian HEK293T cells and purified using affinity chromatography, as previously described [26]. Either PBS or ovalbumin (OVA) in PBS were used as controls in *in vivo* experiments. OVA was Triton-X114 treated as previously described [40, 41] to remove any LPS from the commercial product prior to use *in vivo*.

### Continuous infusion via osmotic minipump

ALZET osmotic minipumps (supplied by Charles River UK) of 100 μl capacity were selected according to the duration of infusion required for individual experiments (model 1002—14 days; model 1004—28 days). In a sterile Class II hood minipumps were filled with the substance for infusion (sterile HES, sterile TGM, or sterile PBS/OVA protein control) and primed overnight by incubation in sterile PBS at 37°C. Under general anaesthesia (isoflurane) abdominal fur was removed by shaving and the skin was prepared with chlorhexidine solution (Hydrex Clear). The peritoneal cavity was accessed through an upper midline incision and the minipump was placed in the right paracolic gutter. Closure was in two layers with 5-0 undyed Vicryl (Ethicon UK).

### Purification of naïve CD4+ T cells from Foxp3-reporter mice

Naïve CD4^+^ T cells required for the induction of the T cell transfer model of colitis were purified from the spleen and peripheral lymph nodes of Foxp3-GFP reporter mice. Isolated cells were firstly pre-sorted by magnetically activated cell sorting (MACS) selection for CD4^+^ using a depletion isolation strategy that leaves CD4^+^ cells untouched, performed either on manual LD columns (Miltenyi) or on an AutoMACS Pro Separator (Miltenyi). Cells were then further purified by fluorescence-activated cell sorting (FACS) by staining with anti-CD4-APC (clone RM4-5/BioLegend) and anti-CD25-PE (clone PC61.5/eBioscience) antibodies and then sorted for CD4^+^CD25^–^ Foxp3^–^(GFP) on a BD FACSAria III. Following FACS purification, live cells were counted using 0.4% trypan blue and a haemocytometer. Cells were next washed three times in sterile PBS before finally resuspending in sterile PBS to a concentration of 2.5 × 10^6^ cells/ml. Mice received 200 µl of this suspension (5 × 10^5^ cells) for the induction of T cell transfer colitis.

### Induction and monitoring of colitis

To induce colitis in the DSS model [42, 43], mice were placed on 2% or 5% DSS (36,000—50,000 MW, MP Biochemical) in tap water and allowed to drink *ad libitum* for the duration of the experiment (4–7 days). Mice were monitored and scored daily for the duration of the experiment using a disease activity index (DAI) score matrix (Supplementary Table 1) and graphed as Disease Score vs Days.

To induce colitis in the TNBS model [44], BALB/c mice were anaesthetized using inhalational isoflurane and intrarectally administered with 150 µl of a solution of 2.5% TNBS in 50% ethanol or 50% ethanol alone (control) using flexible tubing (ID 0.75 mm/OD 1.6 mm) attached onto a 1 ml syringe which was inserted 4 cm proximal to the anus. The mouse was held upside down for 1 min after administration to ensure absorption. Mice given HES were intraperitoneally injected with 10 µg of HES in 200 µl PBS on days 0 and 1. Mice were monitored and scored daily for the duration of the experiment using the DAI score matrix (Supplementary Table 1) and graphed as Disease Score vs Days.

For induction of colitis in the T cell transfer model [45, 46], following cell isolation and MACS/FACS purification, 5 × 10^5^ naïve CD4^+^CD25^−^GFP^−^ T cells isolated from the spleen and peripheral lymph nodes of Foxp3-GFP reporter mice. T cells were adoptively transferred by intravenous injection into each RAG1^−/−^ recipient. Mice were monitored regularly and scored for the duration of the experiment using the DAI score matrix (Supplementary Table 1) and graphed as Disease Score vs Days (post cell transfer).

### Colon tissue processing

The colon was firstly dissected from the mouse just below the caecum and then at the rectum, with its length measured using a ruler before being flushed with ice cold PBS (to remove faeces) and having its weight measured using a fine balance. Results were graphed either as a ratio of colon weight/length (mg/cm), or just colon length (mm) if the weight measurement was not taken.

Tissue required for analysis of myeloperoxidase (MPO) activity and tissue cytokines was a 0.5 cm section from the proximal colon, nearest the caecum and adjacent to the tissue used for histology; tissue was dissected, blotted dry, and rapidly frozen in dry ice before storage −80°C until assayed.

### Intestinal tissue processing and histological scoring

Dissected colon and small intestine tissues were washed in cold PBS, inverted onto PBS pre-soaked wooden skewers (brochettes) and semi-fixed in 10% neutral buffered formalin (NBF) solution (Sigma) for 3–5 h before being fully cut longitudinally with a scalpel blade, rolled into so-called ‘Swiss rolls’ [47], placed in double height histology cassettes and fixed in 10% NBF for a further 14–19 h. The fixed tissue rolls were next rinsed in PBS and transferred into 70% ethanol for 24 h before being processed for histology and embedded in paraffin. Cross-sections of 5 μm were cut and stained with haematoxylin and eosin.

Scoring of histological specimens for all T cell transfer experiments was performed in a blinded fashion using a matrix of six parameters: (i) crypt architecture; (ii) ulceration; (iii) crypt abscesses; (iv) goblet cell loss; (v) mucosal inflammatory infiltration; and (vi) submucosal inflammatory infiltration. Each parameter was given a score of 0–3 with the final value being the sum of these, the maximum score being 18. Scoring of histological specimens for DSS experiments was performed in a blinded fashion using a severity of tissue disruption score range of 0–3: 0 = no damage; 3 = severe damage.

### Cellular immunology assays

Single cell suspensions were made from murine mesenteric lymph nodes (MLN) by maceration through 70 μm filters (BD) into complete RPMI 1640 (cRPMI) medium containing HEPES (Gibco), supplemented with 2 mM l-glutamine, 100 U/ml penicillin, and 100 μg/ml streptomycin (Gibco), 10% heat-inactivated foetal calf serum (Gibco). Contaminating red blood cells were removed by resuspending the cells from one spleen in 2 ml of red blood cell lysis buffer (Sigma) and incubating at RT for 2 min. Cells were then washed with cRPMI and counted on a haemocytometer by trypan blue exclusion.

For flow cytometry analysis of murine blood taken at 21 days post naïve T cell transfer, a few drops of blood were collected into several millilitres of FACS buffer containing EDTA (to prevent coagulation), centrifuged for 5 min at 1500 *g* and pellets resuspended in 3 ml of red blood cell lysis buffer (Sigma), incubated for 5 min at room temperature, topped up with FACS buffer and recentrifuged for 5 min at 1500 *g*. Cells were then used for subsequent flow cytometry staining and analysis (see below).

### Flow cytometry staining/analysis

Cells were washed in PBS and stained for viability with LIVE/DEAD® fixable blue (Life Technologies) which was diluted 1:1000 in PBS; 200 μl was added to each sample of cells, which were then incubated in the dark for 20 min at 4°C and washed twice in FACS buffer (PBS containing 0.5% BSA, 5 μM EDTA, and 0.05% sodium azide). To prevent non-specific antigen binding, cells were incubated with either 50 μl of polyclonal IgG (Sigma) or anti-CD16/32 (clone 93; BioLegend) (diluted 1:50 in FACS buffer) for 10 min at 4°C and then washed twice in FACS buffer. Single stain controls were individually added to one drop of UltraComp eBeads (eBioscience). Samples were incubated with a surface stain combination, as indicated in each figure legend, for 20 min at 4°C, washed twice in FACS buffer and then resuspended in 200 μl FACS buffer for acquisition of surface marker data directly, or further processed for intracellular staining. Samples were acquired on a BD Biosciences LSR II or Celesta flow cytometer and analysed using FlowJo software (Tree Star).

### Cytokine measurements

Serum samples analysed for cytokines were taken at the end of experiments by collecting mouse blood into Microtainer serum collection tubes (BD) which were then centrifuged to separate out the serum. Serum was analysed either neat or diluted 1 in 10 and cytokines measured by either Cytometric Bead Array Flex Sets (BD) according to manufacturer’s instructions or standard ELISA protocols for individual cytokines. Results are graphed as pg/ml of sera.

Colon tissue samples analysed for cytokines were resuspended in 400 μl of 2× Cell Lysis Buffer (Cell Signalling) supplemented with 1 mM PMSF (Sigma) and homogenized using a Tissue-Lyzer II (Qiagen) at 30 Hz for 4 min (5 mm metal bead included at time of buffer addition). Samples were then centrifuged at 13,400 *g* for 10 min at 4°C and total protein concentrations of the homogenized tissue supernatant were quantified using the Bradford Assay (samples being measured in duplicate). Colon homogenate supernatant was analysed for cytokines either neat or diluted 1 in 10 and cytokines measured by either Cytometric Bead Array Flex Sets (BD) according to manufacturer’s instructions or standard ELISA protocols for individual cytokines. Results are graphed as pg/mg tissue.

### Determination of tissue MPO activity

Determination of tissue MPO, primarily produced by neutrophils, was based on a previously published protocol [48] and performed by solubilizing MPO with hexadecyltrimethylammonium bromide (HTAB) and then measuring for its activity using a dianisidine-H_2_O_2_ assay. In brief, colon tissue samples taken from the area between the proximal and mid colon (adjacent to the tissue used for histology) were rinsed with cold PBS, blotted dry, and stored at −80°C. For assaying, tissue samples were weighed, suspended in 0.5 ml cold 50 mM potassium phosphate buffer (pH 6.0) containing 0.5% HTAB (Sigma) and homogenized using a Tissue-Lyzer II (Qiagen) at 30 Hz for 4 min (5 mm metal bead included at time of buffer addition). Samples were then centrifuged at 13,400 *g* for 10 min at 4°C. The MPO level in 10 μl of supernatant was determined by adding 190 μl of o-dianisidine dihydrochloride (0.167 mg/ml; Sigma) and H_2_O_2_ solution (0.0005%) and measuring the change in absorbance over 1 min at 450 nm using a 96-well microtitre plate reader. One unit of MPO activity was defined as the quantity able to convert 1 μmol of H_2_O_2_ to water in 1 min at RT and was expressed as units/mg protein. Total protein concentrations of homogenized tissue were quantified using the Bradford Assay. Samples were measured in triplicate.

## Results

### HES dampens the inflammatory response in DSS-induced colitis

**Figure 1:**
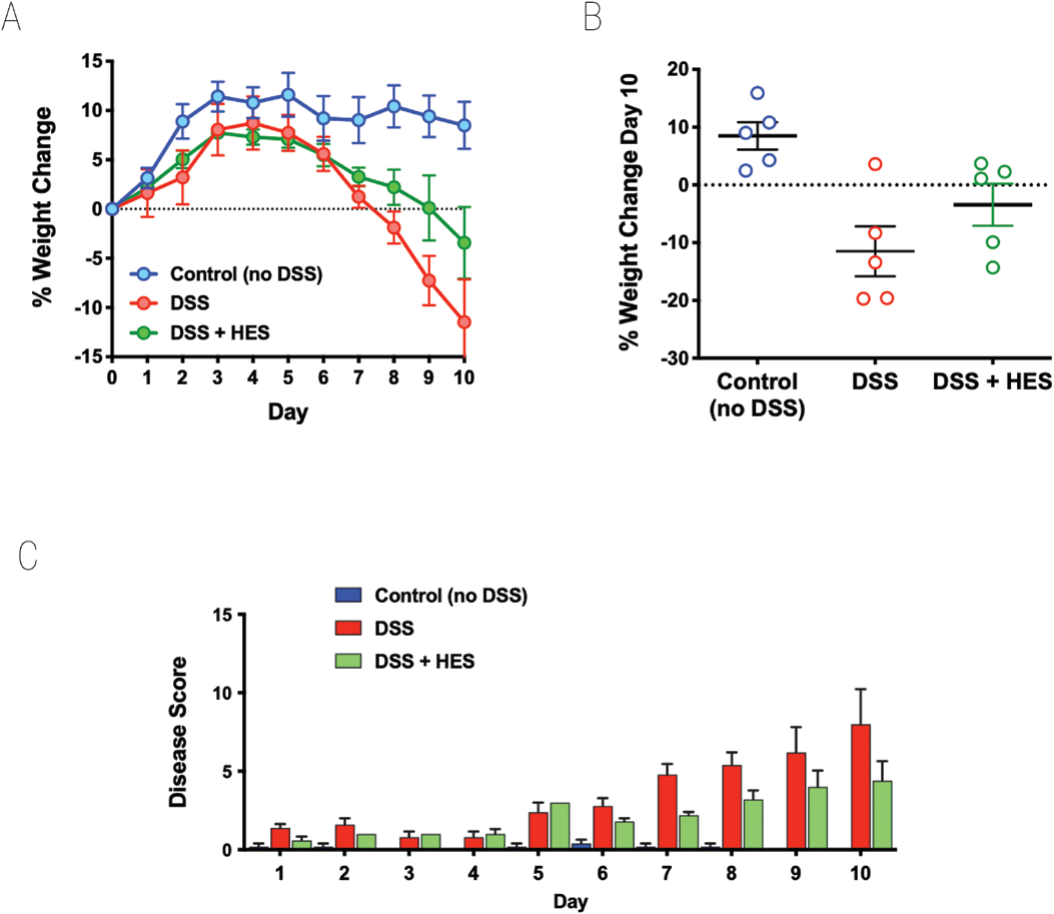
effect of HES in the DSS model of Colitis. DSS was administered as a 2% solution in drinking water to induce acute colitis in C57BL/6 mice for the duration of the 10-day experiment. A 10 µg amount of HES was administered daily from day 0 to day 7 by intraperitoneal injection to one of the DSS groups; the other DSS group received nothing or PBS; a third group (control) received neither DSS nor HES. Results are one of four similar experiments, with *n* = 5 for the DSS groups presented. (**A**) Weight change over time course; data represent means ± SE. (**B**) Comparison of individual weights at day 10; horizontal solid line represents weight at the start of DSS treatment. (**C**) Time course of Disease Scores (Disease Activity Indices) in the three groups.

To assess the effects of *H. polygyrus* HES, we first used the well characterized model of DSS administration to induce acute colitis in C57BL/6 mice [42, 43]. In initial experiments, HES was administered (10 µg/day) from day 0 to day 7 of the study, and mice were monitored over a 10-day period. HES treatment reduced the degree of weight loss, although this did not reach statistical significance (Fig. 1A and B). The overall effects of DSS colitis were evaluated by a DAI matrix that scored body weight, stool consistency and blood content, and motility (see Supplementary Table 1); again HES-treated animals tended to show lower disease scores throughout the experiment (Fig. 1C).

**Figure 2:**
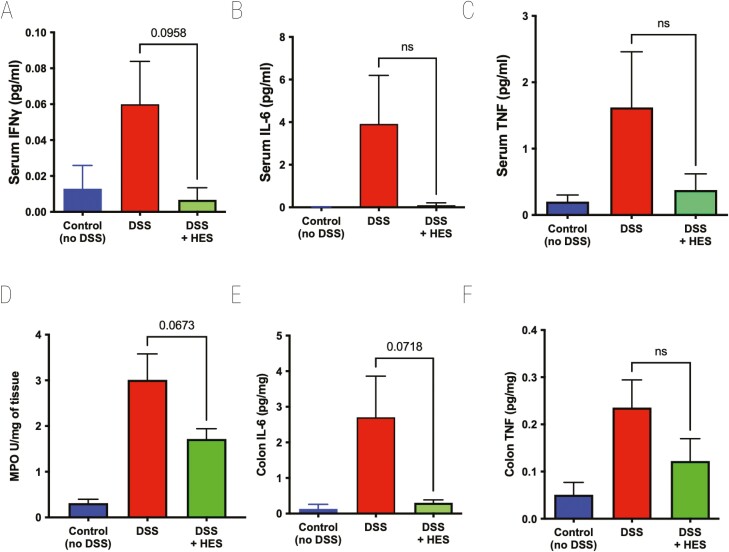
effect of HES on Cytokine levels in DSS Colitis. Serum and colon samples taken from mice at day 10 of DSS treatment, as shown in Fig. 1, for measurement of cytokines by ELISA, and MPO in colon homogenates as detailed in Materials and Methods. Data represented as means ± SE, and *P* values calculated by unpaired *t* tests are shown if <0.1; ns = not significant. (**A**) IFN-γ cytokine levels in serum, (**B**) IL-6 cytokine levels in serum, (**C**) TNF cytokine levels in serum, (**D**) MPO, (**E**) IL-6 cytokine levels in colon homogenate, (**F**) TNF cytokine levels in colon homogenate.

Cytokine levels in serum were measured in samples taken at day 10 of DSS-induced colitis. DSS provoked high levels of IFN-γ, IL-6, and tumour necrosis factor (TNF), each of which were reduced to control levels in mice receiving HES, although effects were statistically marginal at best (Fig. 2A–C). No significant quantities of serum IL-5, IL-10, IL-12p70, IL-13, or IL-17A were detected in any group (data not shown). To evaluate tissue damage in the colon, we measured MPO activity, which was elevated in all DSS-treated animals, although less so in those also receiving HES (Fig. 2D). We also found high levels of IL-6 and TNF in DSS-treated colonic tissue, but these cytokine responses were subdued in HES recipients (Fig. 2E and F). Colonic IFN-γ levels were variable and did not differ between control and DSS groups (data not shown).

**Figure 3:**
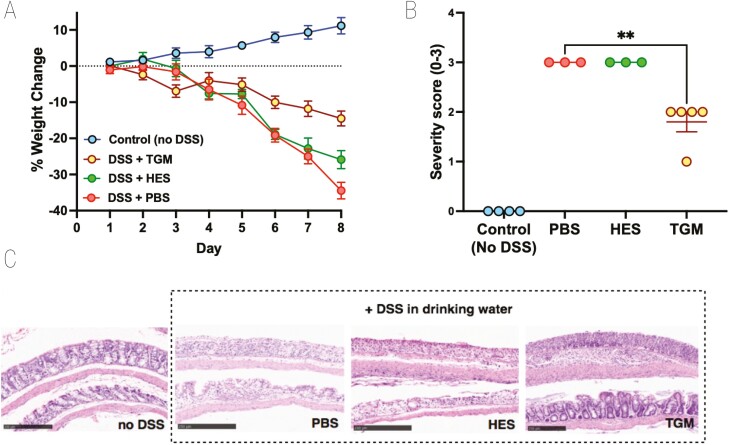
effect of TGM on 4 day model of DSS colitis. Mice were injected intraperitoneally with either PBS, HES (5 µg) or TGM (500 ng) at days 0, 2, 4, 6, and placed on 5% DSS drinking water from day 0 to 3 before switching over from day 4 to normal drinking water for the remainder of the time course (up to 8 days). Mice that were not placed on DSS were injected with PBS as above and were given normal drinking water for the entire experiment. All mice were weighed daily and at the end of the experiment had colons fixed for histology and scored for severity; two individual mice in each of the PBS and HES-treated groups showed >25% weight loss at day 7 and were euthanized at that point; histology was evaluated only on the remaining animals. Female C57BL/6 mice; *n* = 3–5; data shown are from one of two replicate experiments. (**A**) Percentage change from body weight at the start of the experimental period. (**B**) Histopathology scoring, according to the scoring system described in Materials and Methods; statistical analysis by unpaired *t* test; **P < 0.01. (**C**) Examples of histological staining of colon tissues from control mice and those receiving DSS in drinking water with or without administration of HES or TGM. 10× magnification, scale bar represents 250 µm

In additional experiments, we also tested the ability of the recently described TGF-β mimic (TGM) protein from *H. polygyrus*, which has been shown to prolong allograft survival in mice when administered through an osmotic minipump [26]. However, using a similar protocol of 50 ng/day did not show amelioration of DSS colitis (Supplementary Fig. 1A and B), while oral gavage 1 µg/day of TGM was similarly ineffective (Supplementary Fig. 1C and D). In parallel studies, we had succeeded in ameliorating DSS colitis by administering high-oral doses in a curtailed (4-day) regimen [34], while also finding that intraperitoneal injection of TGM strongly suppressed inflammation in an airway allergy model [33]. We therefore tested i.p. administration of HES and TGM in a 4-day DSS protocol and found prolonged protection by TGM as evaluated by reduced weight loss (Fig. 3A) and significantly lower levels of tissue damage when histological sections were evaluated (Fig. 3B and C). As previously, HES restrained DSS-induced colitis to only a minor degree.

We then assessed whether HES could inhibit colitic inflammation in another model, in which TNBS is administered intrarectally to BALB/c mice, provoking a rapid innate inflammatory reaction within 2–3 days [35]. In this system, however, we found HES did not alter outcome measured by weight loss (Supplementary Fig. 2A), colonic shortening (Supplementary Fig. 2B), or DAI Scoring (Supplementary Fig. 2C). Taken together with the results of DSS colitis, we concluded that in these acute models of innate immunity-mediated colitis, while active infection with *H. polygyrus* is known to suppress inflammation [21, 24], inhibition by parasite products at the doses used was too slight to warrant further investigation.

### HES suppresses colitis following adoptive transfer of naïve CD4^+^Foxp3^−^ T cells into RAG1^−/−^ recipients

**Figure 4:**
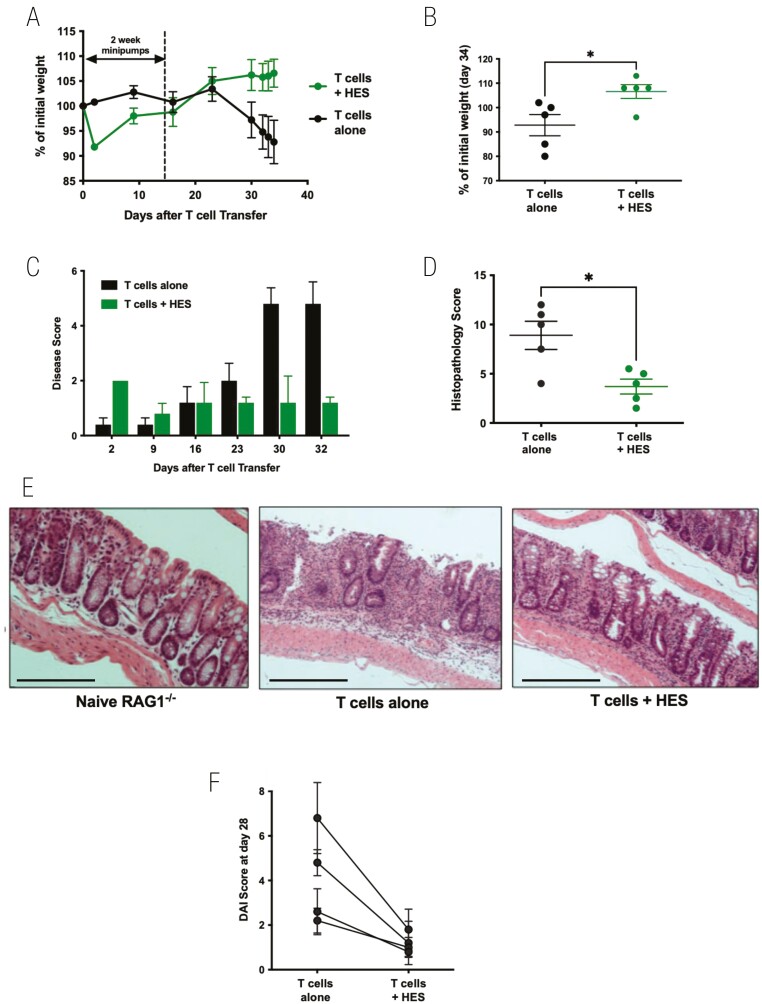
HES suppresses colitis following adoptive transfer of naive CD4^+^Foxp3^−^ T cells into RAG1^−^/^−^ recipients. RAG1^−^/^−^ mice were divided into two experimental groups—receiving either no treatment, or a continuous infusion of HES via an intraperitoneal osmotic minipump eluting 6.8 μg/day for 14 days. One day after minipump implantation, 5 × 10^5^ sorted naïve CD4^+^CD25^−^GFP^−^ T cells from spleens of Foxp3-GFP reporter mice, were adoptively transferred i.v. into each RAG1^−^/^−^ recipient mouse. Results are one of four similar experiments, with *n* = 5 for the groups presented, except for panel F which presents a summary of mouse weights at day 28 post-T cell transfer in all four experiments. Data were analysed by unpaired *t* tests; *P < 0.05. (**A**) Weight change over time course, (**B**) Weight change at day 34, (**C**) Time course of Disease Scores (Disease Activity Indices), (**D**) Histopathology scoring, according to the scoring system described in Materials and Methods, (**E**) Example histological sections (10× magnification, scale bar represents 250 µm), (**F**) Summary of Disease Scores in four independent experiments at day 28 after T cell transfer.

An established model of intestinal immunopathology is a T cell transfer model of colitis, in which lymphocyte-deficient mice seeded with effector T cells develop intestinal inflammation over a 3–6 week period [35, 45, 46]. A series of experiments were undertaken using osmotic minipumps to continuously release HES for periods of 14–28 days, administering totals of 75–100 µg per animal. First, 14-day minipumps were implanted into RAG1^–/–^ mice intraperitoneally, and the following day mice received an i.v. infusion of 5 × 10^5^ naïve CD4^+^CD25^–^Foxp3-GFP^–^ T cells isolated from the spleen and peripheral lymph nodes of Foxp3-GFP reporter mice. Recipient RAG1^–/–^ mice were monitored on a regular basis for 34 days. Beyond day 23, control mice began to lose weight while HES-treated animals maintained or even gained weight (Fig. 4A), a difference which reached significance at day 34 (Fig. 4B). From day 23, control animals began to display signs of disease, while HES recipients showed no deterioration (Fig. 4C). Histopathological analysis of intestinal tissues at day 34 again showed significantly reduced disease scores in the mice given HES (Fig. 4D and E). Peripheral blood from recipient mice taken at day 21 showed similar levels of CD4^+^ T-cell engraftment (Supplementary Fig. 3A) and of Foxp3^+^ expression within the donor T-cell population (Supplementary Fig. 3B); samples collected at the end of the experiment showed that HES tended to reduce serum and colonic IL-6 (Supplementary Fig. 3C and D), and increase IL-10 (Supplementary Fig. 3E), while IL-17 or IFN-γ were maintained (Supplementary Fig. 3F and G).

Similar protection against colitis and weight loss were obtained with a 28-day minipump (not shown). However, only a modest degree of amelioration of weight loss and disease activity was observed when implantation of the minipump was delayed until 14 days post cell transfer (Supplementary Fig. 4). Taking together the four independent experiments in which HES minipumps were implanted the day prior to T cell transfer, a significant reduction (*P* = 0.037) in Disease Score was observed, as presented in Fig. 4F.

### TGM suppresses T cell transfer colitis

**Figure 5:**
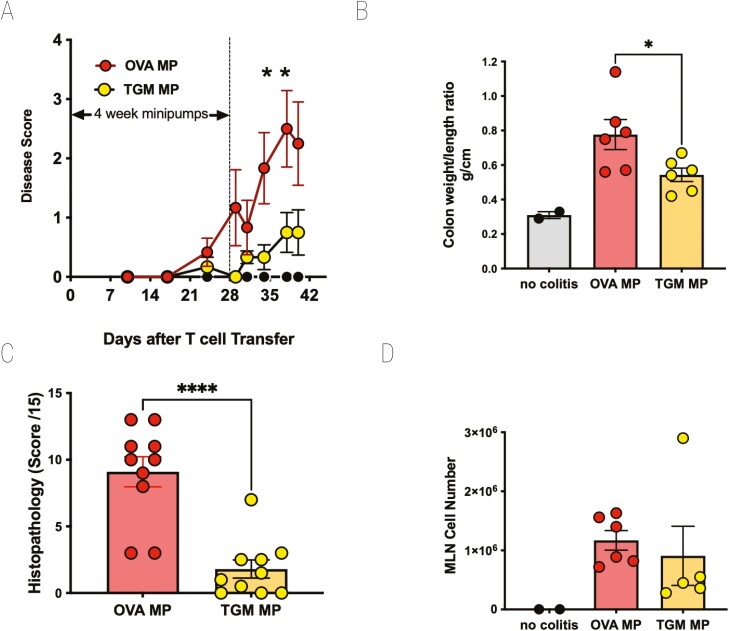
TGM suppresses colitis following adoptive transfer of naive CD4^+^Foxp3^−^ T cells into RAG1^−^/^−^ recipients. As above, CD4^+^CD25^−^GFP^−^ T cells from spleens of Foxp3-GFP reporter mice were adoptively transferred into RAG1^−^/^−^ recipients which then received either no treatment or a 28-day infusion of TGM via an intraperitoneal minipump (eluting 50 ng/day of TGM). (**A**) Percentage change from body weight at the start of the experimental period. (**B**) Colon weight/length ratio. (**C**) Histopathology scoring, according to the scoring system described in Materials and Methods. (**D**) MLN cell numbers. For **A**, **B**, and **D**, results are one of three similar experiments, with *n* = 5–6 for the groups presented. For **C**, data were collected from two experiments each with *n* = 5–6 and pooled. Data were analysed by unpaired *t* tests; *P < 0.05, ****P < 0.0001.

We next evaluated whether a potent immunomodulatory protein within HES, the TGF-β mimic, TGM, could also protect mice from T cell-mediated inflammatory colitis. Using a similar protocol with 4-week infusions from osmotic pumps, RAG1^−/−^-deficient mice receiving CD4^+^CD25^−^Foxp3^−^GFP^−^ T cells were given either 50 ng/day TGM (i.e. 1.4 µg total amount of protein over 28 days) or OVA as a control. Animals receiving TGM were largely protected from weight loss (Fig. 5A), significantly so between days 34 and 38. They also showed less colonic thickening as measured in the colon weight:length ratio (Fig. 5B) and substantially less-histological evidence of inflammation (Fig. 5C). However, we noted that in mice that had received TGM from the day of T cell transfer, the overall engraftment of donor cells appeared to be reduced (Fig. 5D); hence the protection against inflammation might be due to inhibition of systemic T-cell expansion in the lymphopaenic recipients rather than of effector T cells in the tissues.

### TGM modulates colitis following onset of symptoms

To test whether TGM could in fact suppress inflammation, rather than T-cell engraftment following transfer to RAG1^−/−^ mice, we therefore repeated these experiments, but delayed administration of TGM until colitic symptoms had become apparent. Towards the end of week 3, animals began to show one or more signs of disease, and at this time one group of mice received TGM and the others received OVA. As the animals were already symptomatic, daily intraperitoneal injection was preferred over surgical implantation of osmotic minipumps, and 1 µg of TGM or OVA was administered in PBS each day from day 18 to day 29.

**Figure 6:**
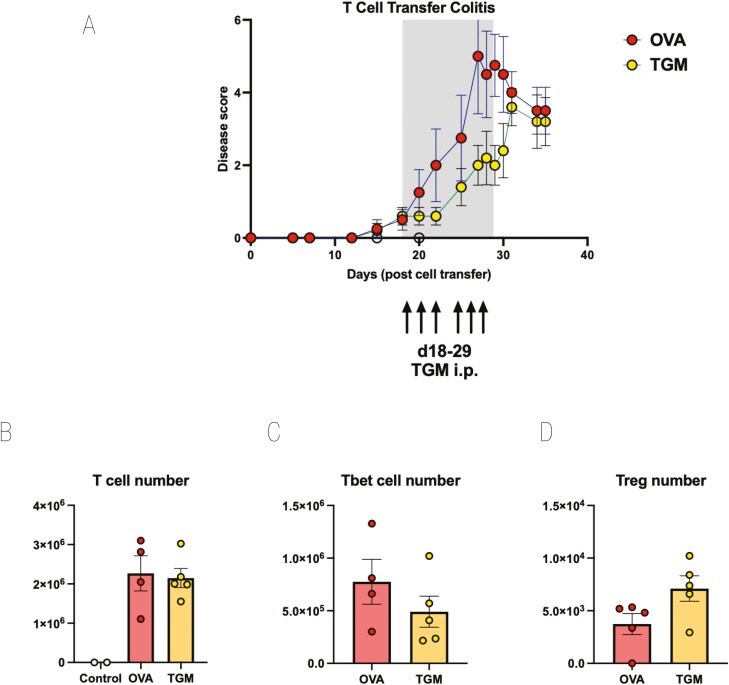
TGM ameliorates disease if given at the onset of symptoms in T cell-mediated colitis. CD4^+^CD25^−^GFP^−^ T cells from spleens of Foxp3-GFP reporter mice were adoptively transferred i.v. into RAG1^−/−^ recipients. Mice were allowed to progress to showing first signs of mild colitis (at day 18). Mice then received either OVA or TGM via six intraperitoneal injections of 1 µg between days 18 and 29. Results are one of two similar experiments, with *n* = 5 for the groups presented. (**A**) Time course of Disease Scores (Disease Activity Indices), (**B**) MLN T-cell numbers, (**C**) MLN Tbet+ T-cell numbers, (**D**) MLN Foxp3+ T-cell numbers. For C and D, single cell suspensions of MLNs prepared as described in the Materials and Methods were stained for flow cytometry analysis using a panel of antibodies comprising CD3-BV711 (clone 17A2; Biolegend), CD4-FITC (clone GK1.5; Biolegend), and CD25-BV650 (clone PC61; Biolegend) followed by intracellular staining using the FoxP3-transcription Staining set (Invitrogen) for Foxp3-ef450 (clone FJK-16s, eBioscience) and Tbet-PerCp-Cy5.5 (clone 4B10; Biolegend).

As shown in Fig. 6A, disease progression was delayed in recipients of TGM with disease scores reduced to a significant degree at day 29, the day of the final administration of TGM. After treatment ceased, the protective effects were lost, and the TGM-treated group was not significantly different from OVA-treated animals. At the endpoint, MLN cells were recovered, showing equivalent T-cell repopulation in both groups (Fig. 6B), although following TGM treatment there was a trend for fewer Tbet^+^ Th1 cells (Fig. 6C) and a greater number of Foxp3^+^ Tregs (Fig. 6D).

## Discussion

Inflammatory bowel disease is increasing in prevalence in many parts of the world with few new therapeutic remedies available [49]. While evidence from both human and laboratory settings indicate that helminth parasites can ameliorate intestinal inflammation, it is important to identify specific molecules and mediators that may be able to counter disease. In this report, we show that *H. polygyrus* TGM, a secreted mimic of mammalian TGF-β, can significantly dampen T cell-mediated colitis, reducing disease score and shifting the balance between Th17 and Treg populations. We have recently reported that TGM can exert its effects systemically, in suppressing airway allergy when delivered intraperitoneally [33]. We now find that intraperitoneal TGM administration can also suppress intestinal inflammation, in addition to our earlier report in which high doses of TGM delivered in drinking water attenuated DSS-induced colitis [34].

*H. polygyrus* is not unique in ameliorating intestinal inflammation. ES from the human hookworm *Ancylostoma caninum*, given to mice daily i.p. during chemically induced colitis, has been shown to exert a modest reduction in pathological scores as well as the inflammatory cytokines, IFN-γ, and IL-17 [50], and also to raise IL-4 and IL-10 responses [51]. Extracellular vesicles within helminth ES products have been found to ameliorate colitic pathology [52–54], but to develop novel therapies many investigators have isolated defined molecular products. Thus, an immunomodulatory protein in the ES of *A. caninum* was identified as the hookworm anti-inflammatory protein-1 that blocked TNBS-induced colitis [55] and a homologous product from the human hookworm *N. americanus* protected against T cell transfer-mediated colitis [56]. Other helminth products with reported anti-colitic activity include cystatins from the filarial nematode *Brugia malayi* [57] and the trematode *Clonorchis sinensis* [58], and a serine protease from *Trichinella spiralis* [59]. An interesting development has been to express another helminth cystatin, from *Acanthocheilonema viteae*, in a modified probiotic *E. coli* strain, that protects mice against DSS colitis [60]. In addition, modest attenuation of DSS colitis was reported with a peptide from *Schistosoma japonicum* [61] while the glutathione-*S*-transferase from *Schistosoma haematobium*, P28GST, reduces TNBS-induced colitic inflammation in mice [62, 63] and has entered trials in Crohn’s disease patients [64].

Mouse models are invaluable for indicating the likely mechanisms by which helminths and their products can inhibit colitis. *H. polygyrus* can generate dendritic cells (DCs) that confer protection in a T cell transfer model of colitis [65], while DCs exposed to the tapeworm *Hymenolepis diminuta* can protect recipients against DNBS-induced colitis [66]. Many different helminths have been reported to modulate macrophages towards an anti-colitic state [67–70], and helminth-conditioned macrophages can activate CD25^+^ T cells to suppress colitis [71]. Studies are now under way to establish the effects of TGM on DCs and macrophages, two populations known to be heavily influenced by TGF-β signalling [72].

TGM, like TGF-β, is a powerful inducer of Foxp3^+^ Tregs *in vitro* [25, 28], and the role of Tregs in the control of intestinal inflammation is very well established [73]. However, in our *in vivo* studies, changes to the Treg compartment were relatively modest. Our finding that protection against T cell-mediated colitis waned once TGM administration ended (Fig. 6) might suggest that it acts directly on effector cells (Th17 or M1 macrophages) rather than indirectly through induction of Tregs, as the latter may be expected to have longer-lasting effects. This question is similarly under further investigation.

In our previous report, high doses of TGM delivered in drinking water were able to dampen DSS colitis, albeit in a moderated model with shorter DSS exposure [34]; in the current study, parenteral TGM (at lower doses) was similarly effective, although low-dose gavage or osmotic pump modes of administration could not ameliorate the effects of a full-course DSS model. While these disparities emphasises the importance of ensuring appropriate dose and delivery, the results confirm a striking protective effect of TGM in acute colitis. Moreover, we show that at low doses TGM is also protective in the T cell transfer model of colitis, which, since the T cell transfer model is considered to more closely reflect IBD in humans, indicates promising possibilities for the treatment of intestinal inflammation. Further understanding of the targets of TGM immune modulation *in vivo*, and finer definition of the optimal conditions for its delivery, will be required to realize this potential as a novel therapeutic for intestinal inflammatory disorders.

## Supplementary Material

kyad001_suppl_Supplementary_Figure_S1

kyad001_suppl_Supplementary_Figure_S2

kyad001_suppl_Supplementary_Figure_S3

kyad001_suppl_Supplementary_Figure_S4

kyad001_suppl_Supplementary_Material

## Data Availability

All data presented in this manuscript are available from the author in the form of Excel and Prism files, and original photomicrographs.

## Abbreviations

DAI: disease activity index
DSS: dextran sodium sulphate
GFP: green fluorescent protein
HES: *Heligmosomoides polygrus* excretory/secretory products
HTAB: hexadecyltrimethylammonium bromide
IBD: inflammatory bowel disease
IFN: interferon
MLN: mesenteric lymph node
MPO: myeloperoxidase
NBF: neutral buffered formalin
OVA: ovalbumin
PBS: phosphate-buffered saline
RAG: recombination activation gene
TGF-β: transforming growth factor-β
TGM: transforming growth factor mimic
TNBS: trinitrobenzene sulphonic acid
TNF: tumour necrosis factor
