# Supplementary figures and images for: Protection from T cell-dependent colitis by the helminth-derived immunomodulatory mimic of transforming growth factor-β, Hp-TGM
Source: Discov Immunol. 2023 Jan 18;2(1):kyad001. doi: 10.1093/discim/kyad001 (PMC9958376; doi:10.1093/discim/kyad001)

## Slide 1
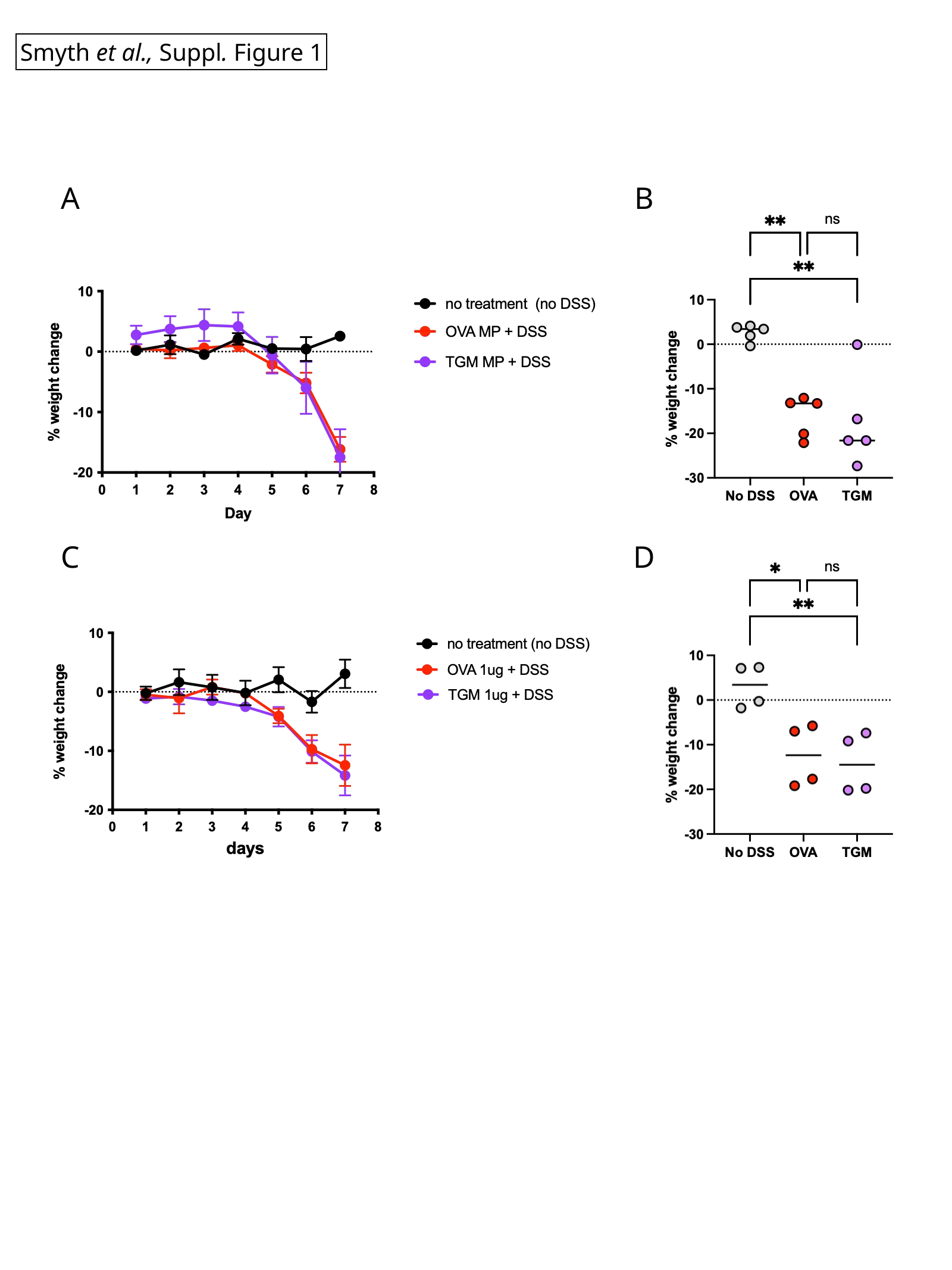

Smyth et al., Suppl. Figure 1
A
B
C
D

Supplement: kyad001_suppl_Supplementary_Figure_S1 [file kyad001_suppl_Supplementary_Figure_S1.pptx]

## Slide 1
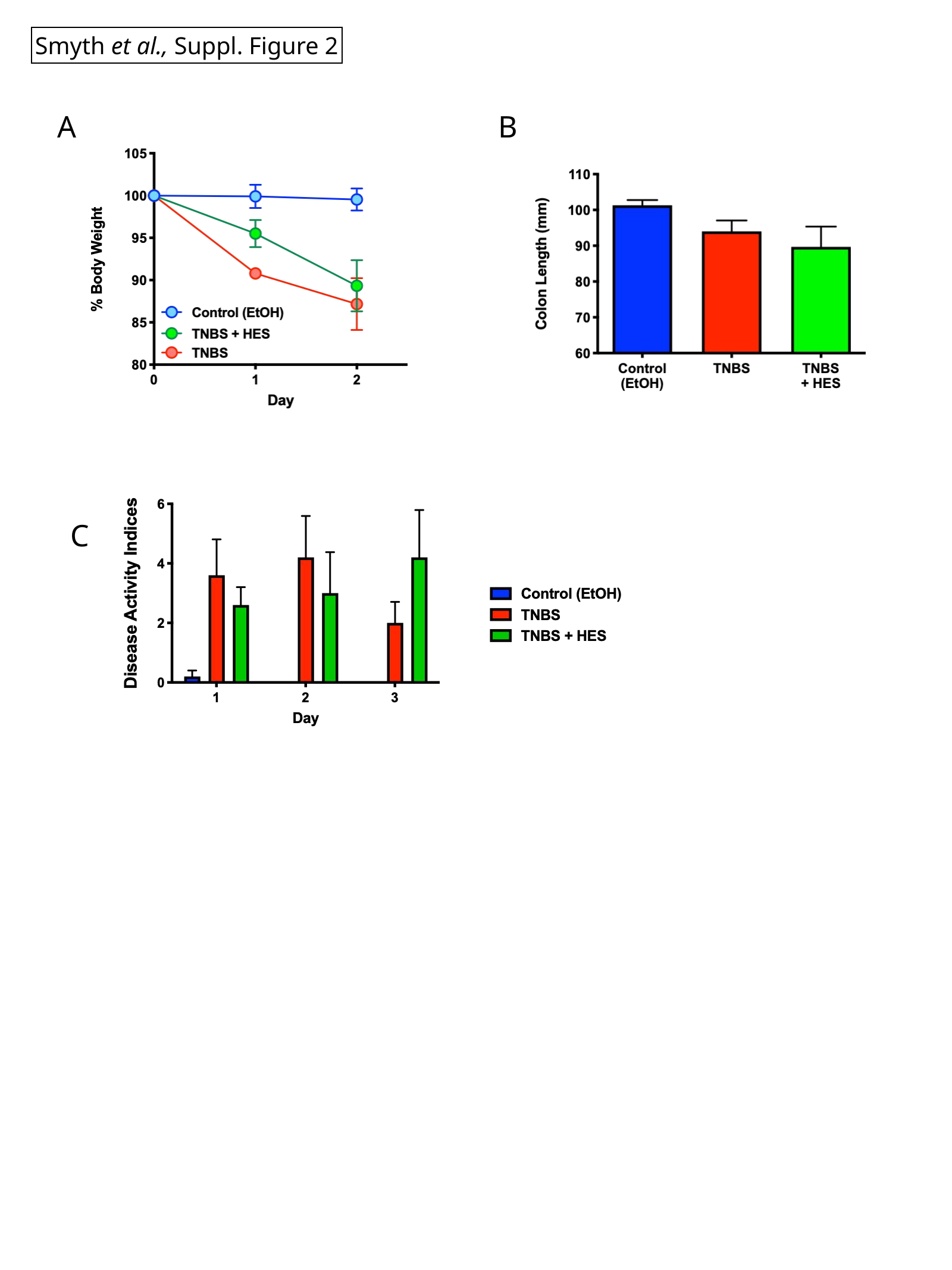

Smyth et al., Suppl. Figure 2
A
B
C

Supplement: kyad001_suppl_Supplementary_Figure_S2 [file kyad001_suppl_Supplementary_Figure_S2.pptx]

## Slide 1
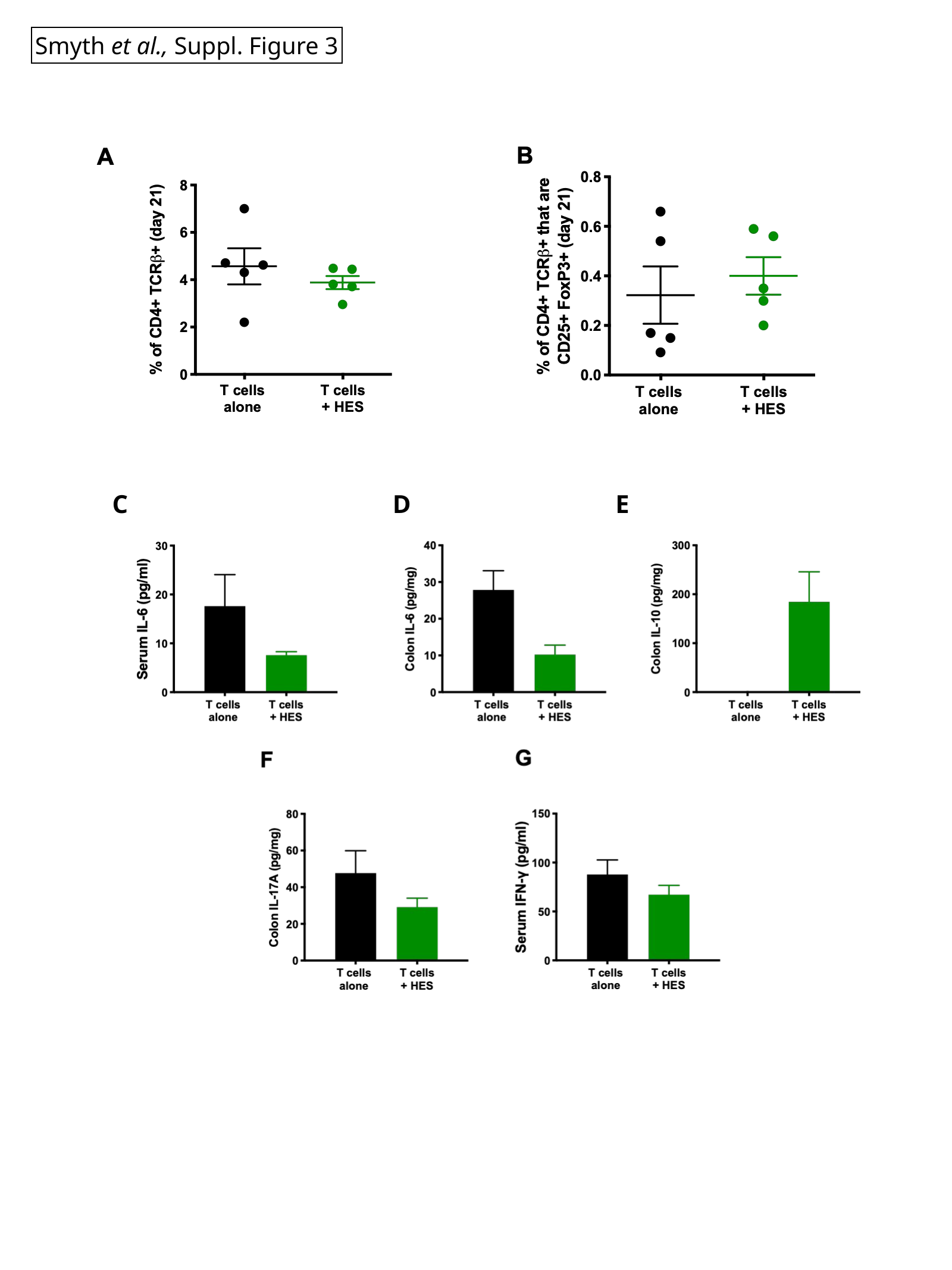

Smyth et al., Suppl. Figure 3
C
D
E

Supplement: kyad001_suppl_Supplementary_Figure_S3 [file kyad001_suppl_Supplementary_Figure_S3.pptx]

## Slide 1
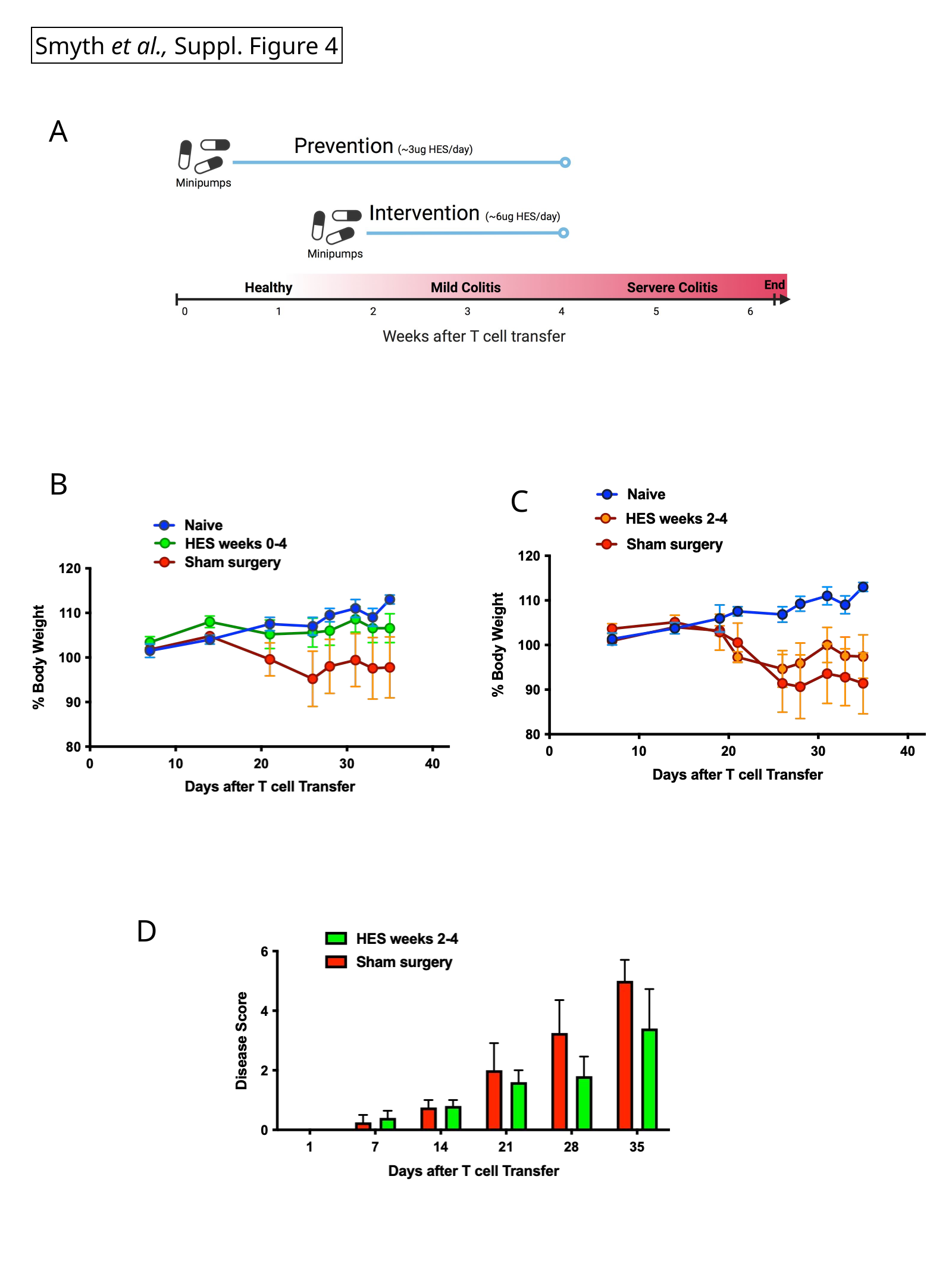

Smyth et al., Suppl. Figure 4
A
B
C
D

Supplement: kyad001_suppl_Supplementary_Figure_S4 [file kyad001_suppl_Supplementary_Figure_S4.pptx]
