# Supplementary material for: Protection from T cell-dependent colitis by the helminth-derived immunomodulatory mimic of transforming growth factor-β, Hp-TGM
Source: Discov Immunol. 2023 Jan 18;2(1):kyad001. doi: 10.1093/discim/kyad001 (PMC9958376; doi:10.1093/discim/kyad001)
Supplement: kyad001_suppl_Supplementary_Material [file kyad001_suppl_Supplementary_Material.docx]

**Supplementary Information**

**Supplementary Figure 1. Effect of TGM in 7-day DSS model of colitis**

Dextran sodium sulphate (DSS) was administered as a 2% solution in drinking water to induce acute colitis in C57BL/6 mice for the duration of the 7-day experiment. Data from two independent experiments delivering TGM by osmotic minipump infusing 50 ng TGM per day (**A, B**); and oral gavage of 1 µg TGM or OVA in PBS per day (**C, D**). For A, B n=5; for C, D n=4. Female mice were used in both experiments. B, D data from day 7 for statistical comparisons using ordinary one-way ANOVA and Tukey’s multiple comparisons test.

**Supplementary Figure 2**. **Effect of HES in TNBS model of colitis.**

BALB/c male mice were given 2.5% TNBS in 50% ethanol intrarectally, with or without 5 µg HES injected intraperitoneally. Data in A and B are from one representative experiment of two; data in C are from a single experiment. Group sizes of 5 mice were used in each experiment.

1. Weight loss
2. Colon length
3. Time course of Disease Scores (Disease Activity Indices)

**Supplementary Figure 3. Effect of HES in T cell transfer model of colitis.**

Blood samples were taken at day 21 post CD4^+^CD25^–^GFP^–^ T cell transfer to measure T cell engraftment (A, B) and cytokines measured in serum or colon homogenate at the end of the experimental model timepoint (day 34). For flow cytometry, red blood cells were lysed using RBC Lysis buffer (Sigma), and lymphocytes stained with the following antibody panel: CD4-APC (clone RM4-5; Biolegend), CD45-PE-Cy7 (clone 30-F11; Biolegend), TCRβ-Pacific Blue (clone H57-597; Biolegend), CD25-PE (clone PE-61.5; eBioscience), and analysed together with Foxp3-GFP from the transferred reporter donor mice.

Cytokines were measured by ELISA with data represented as means ± SE. Male RAG1^-/-^ mice were used in group sizes of 5. Data are from a single experimental set.

1. Percentage of CD4^+^ TCRβ^+^ in blood at Day 21 post naïve T cell transfer
2. Percentage of CD4^+^ TCRβ^+^ that are CD25^+^ Foxp3^+^ in blood at Day 21 post naïve T cell transfer
3. IL-6 cytokine levels in serum
4. IL-6 cytokine levels in colon homogenate
5. IL-10 cytokine levels in colon homogenate
6. IL-17 cytokine levels in colon homogenate
7. IFN-γ cytokine levels in serum

**Supplementary Figure 4. HES in Preventative and Intervention timings using the T cell transfer model.**

Comparison of HES infused minipumps for either weeks 0 - 4 (Preventative), or weeks 2-4 (Intervention). Data are from a single experimental set.

1. Schematic of model (created with BioRender.com)
2. Effects on mouse body weight of preventative HES for weeks 0 - 4
3. Effects on mouse body weight of intervention HES for weeks 2 - 4
4. Effects of intervention HES for weeks 2 - 4 measured by time course of Disease Scores (Disease Activity Indices)

**Supplementary Table 1: Disease Scoring**

For all models of colitis, mice were monitored and accorded a Disease Activity Index (DAI) score (body weight, blood, stool consistency and general appearance) to aid objective comparison of the clinical progression of disease. Scores for each parameter are summed to give a DAI out of a maximum of 16.

| Body weight | Blood | Stool Consistency | General Appearance |
| --- | --- | --- | --- |
| No weight loss (<1%) = 0 | No blood = 0 | Well formed/normal = 0 | Normal = 0 |
| 1-5% weight loss = 1 | Blood present in/on faeces = 1 | Pasty/semi-formed = 1 | Piloerection only = 1 |
| 5-10% weight loss = 2 | Visible blood in rectum = 2 | Pasty/some blood = 2 | Lethargy, piloerection = 2 |
| 10-20% weight loss = 3 | Visible blood on fur = 4 | Diarrhoea that does not adhere to anus = 3 | Motionless, sickly, sunken-eyed, ataxic = 4 |
| >20% weight loss = 4 |  | Diarrhoea that does adhere to anus = 4 |  |
